# Bending Properties of an Extensile Fluidic Artificial Muscle

**DOI:** 10.3389/frobt.2022.804095

**Published:** 2022-04-13

**Authors:** Jacek Garbulinski, Norman M. Wereley

**Affiliations:** Composites Research Laboratory, Department of Aerospace Engineering, University of Maryland, College Park, MD, United States

**Keywords:** soft robotics, soft actuator, fluidic artificial muscles, bending properties, elastic rod model, pneumatic artificial muscles, pneumatic muscle actuator, continuum robot

## Abstract

Low stiffness, large stroke, and axial force capabilities make Extensile Fluidic Artificial Muscles (EFAMs) a feasible soft actuator for continuum soft robots. EFAMs can be used to construct soft actuated structures that feature large deformation and enable soft robots to access large effective workspaces. Although FAM axial properties have been well studied, their bending behavior is not well characterized in the literature. Static and dynamic bending properties of a cantilevered EFAM specimen were investigated over a pressure range of 5–100 psi. The static properties were then estimated using an Euler-Bernoulli beam model and discrete elastic rod models. The experiments provided data for the determination of bending stiffness, damping ratio, and natural frequency of the tested specimen. The bending stiffness and the damping ratio were found to change fourfold over the pressure range. Experimentally validated bending properties of the EFAM presented insights into structural and control considerations of soft robots. Future work will utilize the data and models obtained in this study to predict the behavior of an EFAM-actuated continuum robot carrying payloads.

## Introduction

FAMs are a popular choice for soft robotic systems due to their force output, low weight and manufacturing simplicity. The muscle is a form of soft actuation as its stiffness is magnitudes lower when compared to traditional pneumatic cylinders or linear electric motors (Majidi (2014)). Modeling and testing of their axial behavior has been covered by a number of research groups (Chou and Hannaford (1996); Tondu and Lopez (2000); Takosoglu et al. (2016); Salahuddin et al. (2021); Chambers and Wereley (2021)). Achieving variable stiffness of the actuators was investigated in Xiang et al. (2016). Some research groups also investigated the bending behavior for developing elephant-trunk-like manipulators (Guan et al. (2020)) or to achieve larger contraction ratios (Bruder and Wood (2022)). Buckling behavior was also investigated by Kim et al. (2021).

**FIGURE 1:**
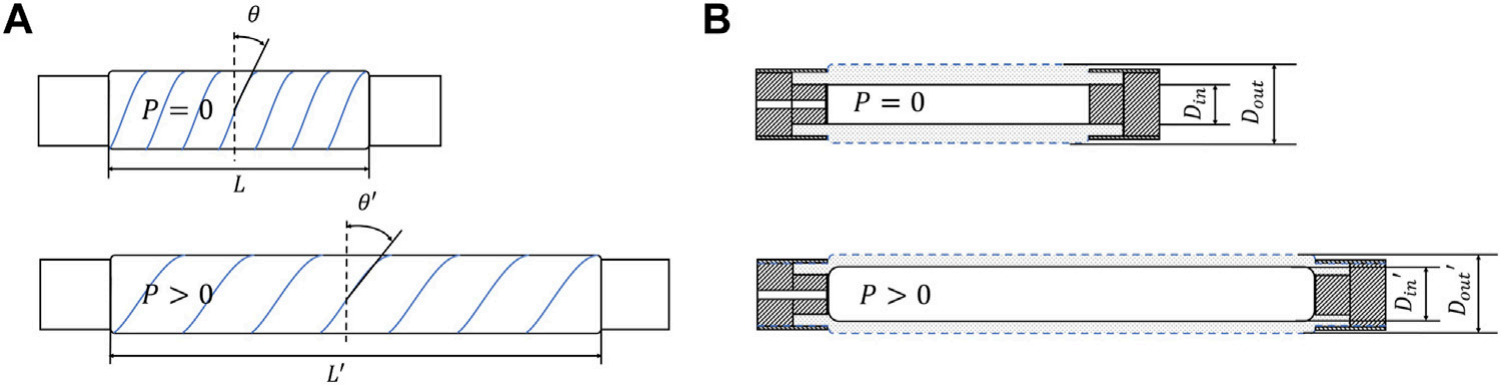
**(A)** A view of an extensile FAM in: a resting state (top), a pressurized state (bottom). **(B)** A longitudinal cross-section view of an extensile FAM in: a resting state (top), a pressurized state without external forces (bottom).

FAMs can be classified as contractile (pullers) and extensile (pushers) based on their behavior when pressurized. The defining parameter for that behavior is their braid angle which is the angle between FAM lateral cross-section and the fiber direction as was shown in Figure 1. A FAM is contractile if the braid angle is larger than 35.26°, or extensile if the braid angle is smaller than 35.26° (Liu and Rahn (2004)).

Unlike contractile FAMs, which were reported to reach normalized length changes between 24% and 35% for pressures of up to 690 kPa (Pillsbury et al. (2017)), extensile FAMs (EFAMs) can provide much larger strokes that exceed their initial length. Theoretically, 200% extension can be achieved at a braid angle of 22° (Garbulinski et al. (2021)), although in practice stroke values of up to 100% have been reported (McMahan et al. (2006); Garbulinski et al. (2021)). Our motivation to study extensile FAMs stems from their compact size and their large stroke that is beneficial for performance of continuum soft robots (Trivedi et al. (2010)).

Fluidic artificial muscles are composed of three main parts that are shown in Figure 1: a bladder (dotted) which is an elastomeric tube typically made from latex or silicone; a braided bi-axial fiber sleeve (blue line) wound around the bladder, which is made from high-strength material such as kevlar; and two rigid end-fittings that bind the sleeve and the bladder together as well as keep the muscle airtight. Since the internal pressure changes are interchangeably converted into force or extension, the EFAM stiffness varies with the pressure. Moreover, as was shown in this study, due to low stiffness, EFAMs are prone to large lateral deformations under loads.

Modeling bending behavior of EFAMs is critical for continuum soft robots as they move with deformation of their intrinsically actuated structure that is composed of EFAMs. However, the EFAM bending behavior is not well characterized in the literature. The EFAM bending behavior was previously modeled in the context of: FAM-based continuum soft robots (FAM-CSR) (Trivedi et al. (2008)), modeling dynamics of a multi-section FAM-CSR (Godage et al. (2016)), braided continuum manipulators such as STIFF-FLOP (Sadati et al. (2017)), and the capability of decoupling stiffness in a FAM-CSR (Giannaccini et al. (2018)). Olson et al. (2020) used an Euler-Bernoulli beam model to analyze bending of a continuum soft robot, which excluded actuator bending stiffness purported to be a source of error.

The practice in these studies was to experimentally validate analysis by either 1) comparing FAM models to measured axial behavior, or 2) by comparing soft robot models to measurements. Although these approaches were valid for modeling many light-payload FAM-CSRs, incorporating actuator bending stiffness into the analysis and developing a method to measure these values is needed. Thus, the key goal of this study is to measure EFAM bending properties, and to assess how bending properties change with EFAM internal pressure. The bending behavior of an EFAM cantilevered specimen was investigated. A characterization method for bending stiffness is proposed, as well as methods to characterize dynamic properties such as natural frequency and damping ratio.

## Methodology

To assess the properties of an EFAM composed of a latex bladder and a kevlar sleeve, a method to determine static and dynamic properties of an EFAM is proposed.

**FIGURE 2:**
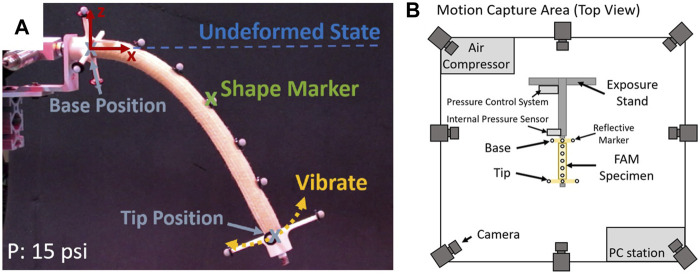
**(A)** A cantilevered extensile fluidic artificial muscle pressurized to 15 psi in a test setup. **(B)** A top view diagram of the test setup.

An experimental setup was developed in which the EFAM was cantilevered and its shape and tip position were tracked with a motion capture system (as shown in Figure 2). For internal pressure values ranging from 5 to 100 psi with five psi increments, the EFAM was horizontally aligned and then released to bend under its own weight. The EFAM was then allowed to stabilize at a static deflection. Markers were sewn to individual fibers along the EFAM to enable tracking with a motion capture system. The movement and the steady-state positions of markers were tracked and recorded by the motion-tracking system. Therefore, the experiment provided us with shape-marker and tip trajectory data.

After collecting experimental data, a static bending beam model was optimized with bending stiffness to match the data in the least-square error sense. For modeling of static bending, a linear Euler beam model and a non-linear planar discrete elastic rod model were used to validate and compare results. Dynamic properties of natural frequency and damping were extracted assuming a second-order system.

### Experimental Characterization

The test setup (shown in Figure 2) consisted of: an EFAM specimen, a Vicon motion capture system, a test stand to expose the markers on the specimen to the motion-capture cameras, a custom closed-loop pressure regulator for maintaining isobaric muscle internal pressure, and a PC computer to record, control, and supervise motion capture.

It was assumed that the motion capture system records the static position of the markers with an error smaller than 0.5 mm and a frequency of 100 Hz. The assumption on the error was found to be conservative when compared with mean absolute error value reported in the literature for a Vicon sytem (Merriaux et al. (2017)). Additionally, the position and rotation of the base was also tracked, and allowed for transformation of the marker global position data into a local frame with origin at the center of the base. The *xz* plane of the local frame was aligned with a vertical plane crossing through a theoretical EFAM center-line. A lab-built closed-loop pressure regulator (Garbulinski et al. (2021)) regulated internal EFAM pressure with a standard deviation smaller than 0.7 kPa.

The Vicon system was first calibrated using the provided calibration wand. The test stand was then placed in the center of the motion capture area so that the markers on the EFAM specimen were well exposed to the tracking cameras. Then, the specimen was covered with a black cloth, and a map of unwanted reflections was created that prevented the Vicon system from identifying the reflections as markers. Then, the markers were uncovered, and the pressurized air system was turned on. After the systems were in place, powered on, initialized and calibrated, the specimen was considered ready for a sub-test at an internal pressure of five psi.

To combat the effects of FAM performance creep (Hocking and Wereley (2012)), at the beginning of each sub-test, the specimen was pressurized 5 times in a cycle from 0 psi to 100 psi and back. After internal pressure cycling, the specimen was pressurized to an actively regulated value of five psi. At this point, length and outer diameter of the specimen were measured with a ruler and a caliper, respectively. After the geometric measurements, the tracking data acquisition was started and directly followed with a start of a handheld timer. Then, the specimen was manually raised to a horizontal configuration and released when the timer indicated that 20 s had elapsed. The FAM was let to vibrate and reach a steady-state in 20 s. After the EFAM reached a steady-state, in a 10 s period the EFAM was raised again to the horizontal position and released. This raise-and-release procedure was repeated 6 times. After the specimen stabilized for the last time, 10 s were added and the tracking was stopped. The acquired data was saved to CSV-format file for post-processing.

The 5-psi sub-test was followed by the same 19 sub-tests with the internal pressure incremented by five psi up to 100 psi. Only increments in pressure were used under the assumption that hysteresis in bending properties was negligibly small. Since the base position was recorded, it was possible to evaluate the position deviation of a tracked object that was assumed to be fixed. With our first iteration of this method, it was observed that the standard deviation of the base position did not exceed 0.19 mm. The error of this size was very close to the tracking resolution of the assumed motion capture system. This assured us that the test stand was rigid during the test and the vibration coming from EFAM dynamic response or manually raising the specimen negligibly affected the results.

### Modeling

With the tip-displacement data acquired through the motion capture testing and a model of beam extension and bending, the flexural stiffness, *EI*, can be determined. Two bending models were applied: an Euler-Bernoulli beam model (Popov (1976)) and a finite-element Planar Discrete Elastic Rod (PDER) model (Goldberg et al. (2019)). The two models enabled comparison of the measured flexural stiffness. The comparison was useful because large EFAM deformations were observed during the experiments. Those deformations had the potential to violate assumptions of the Euler-Bernoulli beam model or induce errors for lower resolution PDER models.

The non-linear model was a discrete and planar approximation of Kirchoff’s rod theory based on the formulation of discrete elastic rod theory developed by Bergou et al. (Bergou et al. (2008)). PDER is a finite element model that for its constitutive equation uses a bending stiffness constant. The theory is sufficiently general to model the stretching, torsional, and flexural deformations of extensible flexible rods. Here, the theory was restricted to the planar case and the torsional deformation was neglected.

Below, two models are presented to calculate bending stiffness from the experimental data, and their results are compared.

**FIGURE 3:**
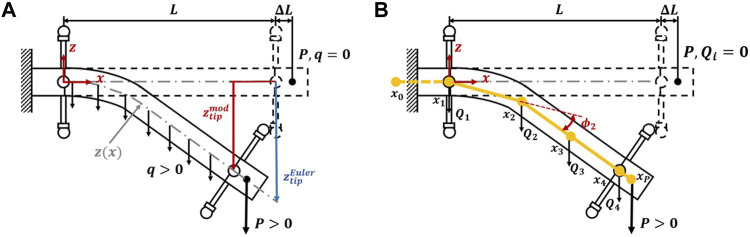
**(A)** Euler-Bernoulli beam model in the context of the experiment. **(B)** PDER model in the context of the experiment.

For beam modeling using a static Euler-Bernoulli model, a super-position method was used. The EFAM was modeled as a beam with a load corresponding to its distributed weight, *q*. It was also subject to a point load, *P*, representing the weight of the end-fitting and the 3D printed marker attachment. The values of *q* and *P* were obtained with weight measurements of specimen parts. A graphical representation of the model was overlaid on a diagram of the experiment in Figure 3. This model gives an equation for the displacement in the vertical coordinate, *z*, as a function of *x* (Popov (1976); Young et al. (2012)):

$$z_{w}\left(x\right)=-\frac{qx^{2}}{24EI}\left(x^{2}+6L^{2}-4Lx\right)$$ (1)

$$z_{F}\left(x\right)=-\frac{Px^{2}}{6EI}\left(3\left(L+\Delta L\right)-x\right)$$ (2)

$$z_{Euler}\left(x\right)=z_{w}\left(x\right)+z_{F}\left(x\right)$$ (3)

where *x* is the horizontal position coordinate and *z* is the vertical position coordinate. A constant, *q* is the distributed load, which is calculated from the weight measurements of the braided sleeve and the bladder. *L* is the active length of the EFAM, measured before each subtest. Δ*L* is a length needed to shift the weight of the end-fitting to the center of the EFAM end tip. *P* is the weight of the EFAM end-tip. *EI* is the bending stiffness of the EFAM. The model assumes negligibly small *x* displacements, which was not the case for the experiment due to low EFAM stiffness. To find the *z* coordinate of the tip, the curve length was numerically integrated along *x* until it was equal the measured length of the EFAM. With that modification, the model gave a more realistic value for *x*_*tip*_ and *z*_*tip*_ (*z*_*tip*_ shown as $z_{tip}^{mod}$ in Figure 3A). A model without this modification (*z*_*tip*_ shown as $z_{tip}^{Euler}$ ) resulted in a relatively large error and was discarded for further analyses.

For the PDER model, the authors refer the reader to references (Bergou et al. (2008); Novelia (2018); Goldberg et al. (2019)) as comprehensive introduction of the model is out of the scope for this paper. In the following model description, only the formulas for elastic energy were presented as they differs from other formulations. A graphical representation of the model for four nodes was shown in Figure 3B. In the figure, the muscle is discretized into *n* − 1 segments of equal length at the resting state with vertices/nodes denoted as **x**_**k**_. In our models, the nodes were equally distributed along the EFAM active length, and their number was set to 4, 8, 16, 32, and 40. A special segment of infinite stiffness, *n*, was also added to represents the end-fitting and the marker cap. Each of the *n* − 1 segments has some weight that comes from the density of the bladder and braided sleeve. Based on those weights and related Voronoi regions, gravitational forces, *Q*_*i*_, are calculated for each node. Each segment, **e** ^**i**^ can rotate with an angle of *ϕ*_*i*_ and corresponding curvatures, *κ*_*k*_. These are given by (Goldberg et al. (2019)):

$$e^{i}=x_{i+1}-x_{i},\;\;\;\kappa_{k}=\frac{2\sin\left(\varphi_{k}\right)}{1+\cos\left(\varphi_{k}\right)}=2\tan\left(\frac{\varphi_{k}}{2}\right).$$ (4)

The elastic energy, *E*_*e*_, of the system is modeled as:

$$\begin{array}{cc} & E_{e}=E_{s}+E_{b},\\ & E_{s}=\frac{1}{2}\overset{n-2}{\underset{j=0}{\sum }}EA^{j}{\left(\frac{\left\|e^{j}\right\|}{\left\|{\bar{e}}^{j}\right\|}-1\right)}^{2}\left\|{\bar{e}}^{j}\right\|,\\ & E_{b}=\frac{1}{2}\overset{n-2}{\underset{i=1}{\sum }}\frac{EI_{i}}{{\bar{\ell }}_{i}}{\left(\kappa_{i}-{\bar{\kappa }}_{i}\right)}^{2}. \end{array}$$ (5)

where *E*_*s*_ is the extensional energy with a stiffness constant *EA* and *E*_*b*_ is the bending energy with a bending constant *EI*.

Data from models of increasing number of nodes showed us how the model error behaved based on discretization resolution. Note that the PDER model requires extensional stiffness which was obtained in prior research Garbulinski et al. (2021). With the PDER model, our aim was to capture the non-linear behavior of EFAM bending. As shown in Goldberg et al. (Goldberg et al. (2019)), other energies and forces can be added to the model and turned into a state-space dynamic model. Here, we only found static solutions of the state-space where the velocities ${\dot{x}}_{k}=0$ and optimized the tip error with the bending stiffness *EI*.

For each internal pressure test data, both models were optimized with a MATLAB non-linear solver, Fminsearch., 2021)), to find the bending stiffness constant that would give the smallest error in the least square sense for the tip displacement data. The fminsearch.m uses the simplex search method of Lagarias et al. (1998).

To find the dynamic properties, the value for the vertical, *z*(*t*), coordinate was extracted from the data. The damped natural frequency, *ω*_*d*_ was found with a Fourier transform of the *z*(*t*) signal. An exponential decay approximation was used to find the damping ratio, *ζ*, from the amplitude decay with the Matlab non-linear solver (Fminsearch., 2021).

$$A\left(t\right)=\frac{A_{0}}{\sqrt{1-\zeta^{2}}}e^{-\zeta \omega_{d}/\sqrt{1-\zeta^{2}}t}$$ (6)

The curve was fit to the consecutive peaks of the *z*(*t*) time histories. Then, the natural frequency, *ω*_*n*_ was calculated with a formula:

$$\omega_{n}=\omega_{d}/\sqrt{1-\zeta^{2}}$$ (7)

## Results

### Data

For some tests, sporadic spurious data points were present in position coordinate values. The data points were diagnosed as the moments when the Vicon system was not able to evaluate marker positions. Except for the spurious data points, no significant difference was observed between the six raise-and-release test data series. For each internal pressure level, only one test without the spurious data points was selected as a representative of given testing conditions.

**FIGURE 4:**
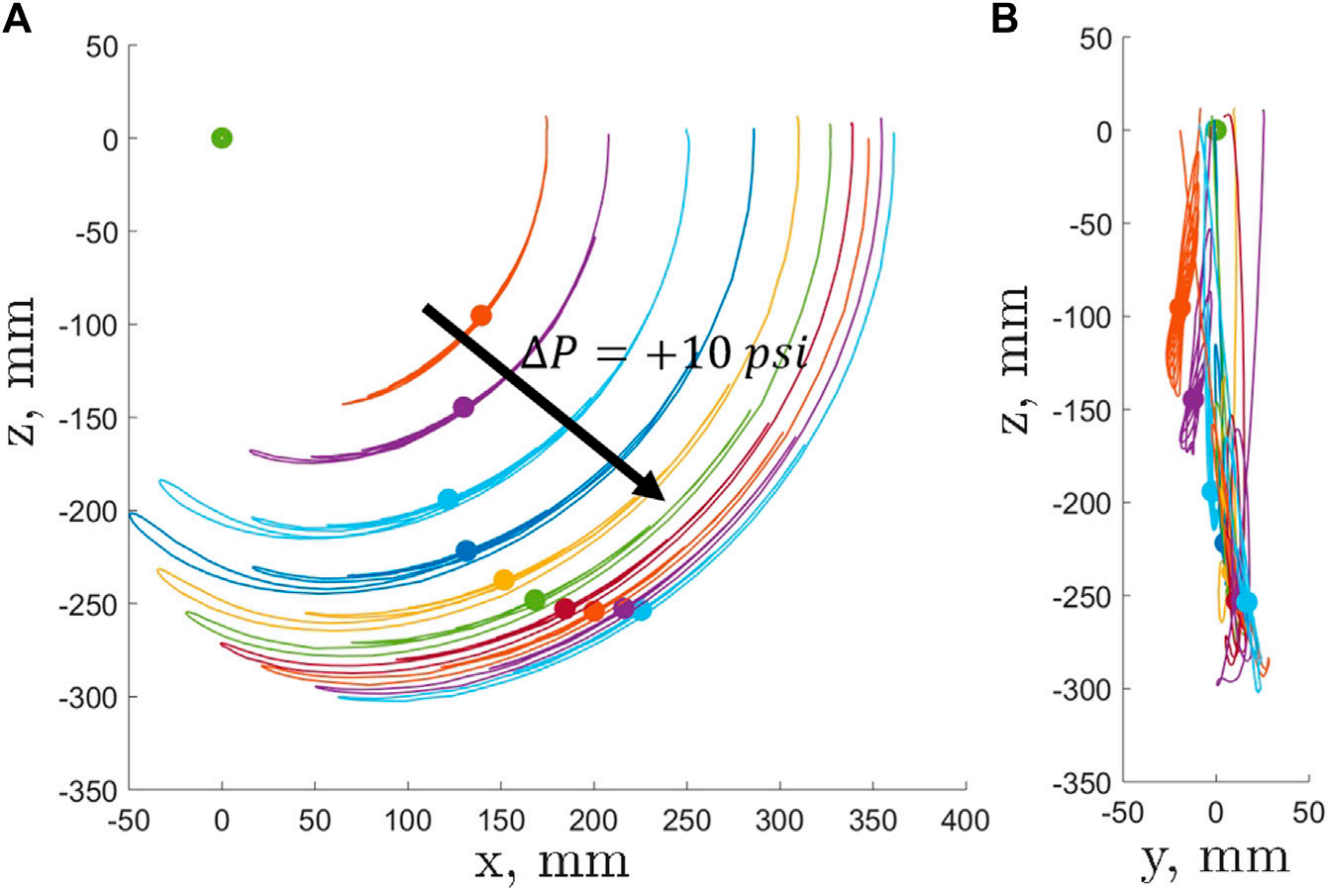
EFAM tip trajectories (solid lines) and static tip positions (circle) in 10 psi increments starting at five psi. The green circle at the axes origin represents position of the base.

An average position of the base was calculated together with average values for the quaternions representing the base rotation in the global coordinate system. Then, the maximum out-of-plane tip deflection for all the recorded trajectories was found to be 35.96 mm. The mean out-of-plane tip deflection of all maximum tip deflections for each case was 20.02 mm with standard deviation of 8.2 mm (Figure 4). The out-of-plane deflections were attributed to the manufacturing imperfections of the bladder which resulted in a specimen that slightly tilted its tip in the lateral directions upon pressurization.

When the lateral deflection was normalized by the length of the EFAM for a given pressure, the worst lateral end-tip deflection was 18.5% of the EFAM length. The mean of the normalized deflection for all pressure levels was 7.12% with a standard deviation of 4%. Keeping in mind a mean error smaller than 10%, only the in-plane position values were used for the further analysis.

In this study, time histories and steady-state tip deflection positions were extracted from the data for each test of different internal pressure level. Tip trajectory and position data was shown in Figure 4. The green dot in the figure represents the position of the base. The figure also shows trajectories of the EFAM tip position which were color-coded for different internal pressures. On each trajectory lies a dot of the same color which represents the steady-state tip position of the EFAM tip. For clarity, only half of the collected data is shown with +10 psi increments starting from the EFAM internal pressure of five psi. One can observe that with increasing pressure the distance to the base increases as the EFAM length increases with pressure. Then, for higher pressures, the length increments are smaller and the EFAM stiffens which changes the steady-state position of the tip.

### Bending Stiffness

**FIGURE 5:**
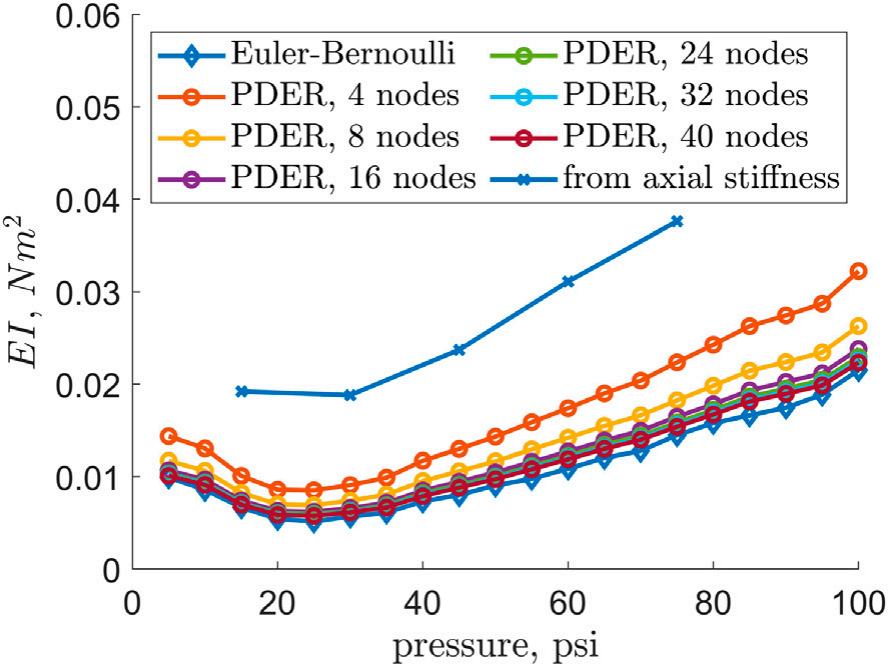
Bending stiffness of an EFAM versus EFAM internal pressure. The blue line represents bending stiffness recalculated from axial rigidity experimental data. The red and yellow lines represent bending stiffness obtained through tip error minimization procedure for PDER models and Euler-Bernoulli models, respectively.

Because of EFAM stiffness changes with the internal pressure, its bending stiffness was expected to change over the pressure range of 5–100 psi. Figure 5 shows the bending stiffness, *EI*, of the EFAM calculated using different methods. The first method with its results was represented with the solid blue line. This method used the axial force data from Garbulinski et al, (2021). The axial rigidity was recalculated to bending stiffness under the assumption that the EFAM was a rod made from isotropic material. For calculation of the bending stiffness, the following formula was used:

$$EI=E\pi \frac{D^{4}-d^{4}}{64}=EA\frac{D^{2}+d^{2}}{16}$$ (8)

where *E* was the Young’s modulus; *A* was the circular cross-sectional area of the EFAM; *D* was the outer diameter measured with a caliper around the center of the EFAM, and *d* was the inner diameter calculated from the EFAM braided sleeve kinematics and an assumption of bladder incompressibility.

**FIGURE 6:**
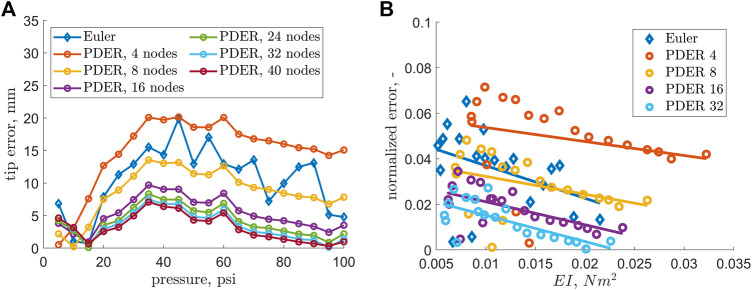
**(A)** Tip displacement error as a norm of Cartesian coordinates relative to the experimental tip positions. **(B)** Tip displacement error normalized by EFAM length as a function of bending stiffness. The markers represent data points, and the solid lines represent least squares linear fit to the data points. Only four out of six PDER models are shown for clarity.

The two methods which results were showed in Figure 5 were described in the previous section, *Modeling*. The figure demonstrated that the values estimated from the optimization were different from those obtained with the axial stiffness data. The values of the Euler-Bernoulli beam model and the PDER models (from 8–40 nodes) were found to be in agreement. The tip error found by the minimization procedure was shown in Figure 6A. Figure 6B also shows that the accuracy of the model improves with increasing bending stiffness of the specimen as well as that the error decreases with the number of nodes in the PDER model.

The numerical data for the modeling was shown in Table 1 and Table 2. In the tables, the subscript 0 indicates the resting state. *θ* stands for the braid angle. *L* stands for the EFAM length. *D*_*in*_ and *D*_*out*_ represent the inner and outer bladder diameter, respectively. *ρ*_*L*0_ is the mass of the active part of the bladder and the braid per unit length. Δ*L* is the length from the end of the bladder to the center of mass of the tip.

**TABLE 1:** Modeling constants.

*θ*_0_, °	*L*_0_, *in*	*D*_*in*0_, *in*	*D*_*out*0_, *in*	*ρ*_*L*0_, *kg*/*m*	Δ*L*, *in*	tip-load mass, *g*	gravity, *m*/*s* ^2^
12.5	6.69	0.97	1.07	0.14227	0.662	54.92	9.81

**TABLE 2:** Modeling variables.

*P*, *psi*	*L*, *in*	*D*_*in*_, *in*	*D*_*out*_, *in*	*EI* (Axial), *Nm* ^2^	*EI* (Euler), *Nm* ^2^	*EI* (PDER 32), *Nm* ^2^
5	6.69	0.97	1.07	0.00601	0.00991	0.01022
10	7.21	0.98	1.07	0.00859	0.00859	0.00923
15	7.99	0.98	1.06	0.01112	0.00667	0.00709
20	8.87	0.98	1.05	0.01355	0.00542	0.00595
25	9.69	0.97	1.04	0.0159	0.00517	0.00582
30	10.41	0.97	1.03	0.01818	0.00568	0.00621
35	11.06	0.96	1.02	0.02038	0.00611	0.00676
40	11.59	0.96	1.02	0.02255	0.00733	0.00801
45	12.01	0.95	1.01	0.0247	0.00803	0.0089
50	12.37	0.95	1	0.02683	0.00905	0.00985
55	12.67	0.95	1	0.02895	0.00977	0.01095
60	12.95	0.94	1	0.03105	0.01087	0.01202
65	13.15	0.94	0.99	0.03317	0.01202	0.01315
70	13.34	0.94	0.99	0.03527	0.01279	0.01414
75	13.51	0.94	0.99	0.03738	0.01448	0.01555
80	13.67	0.93	0.98	0.03945	0.01578	0.01689
85	13.81	0.93	0.98	0.04154	0.01662	0.01833
90	13.93	0.93	0.98	0.04363	0.01745	0.01914
95	14.03	0.93	0.98	0.04573	0.01886	0.0201
100	14.13	0.93	0.98	0.0478	0.02151	0.02259

Two models were used to obtain the bending stiffness values from experimental data and cross-validate the methods. Using the static tip position and optimization techniques, bending stiffness values, *EI*, were found for both models. The discrete element model, the PDER model, led to a trend that was in agreement with the trend obtained with the Euler-Bernoulli beam model (Figure 5). This means that the simpler Euler-Bernoulli can be used to determine bending properties of EFAMs. Another implication of this result is that the values obtained with the Euler-Bernoulli model could be employed in the finite-element PDER models. This is beneficial because the PDER models are useful for simulations of soft robots where modeling of EFAM deformations needs computational approach.

### Shape Comparison

**FIGURE 7:**
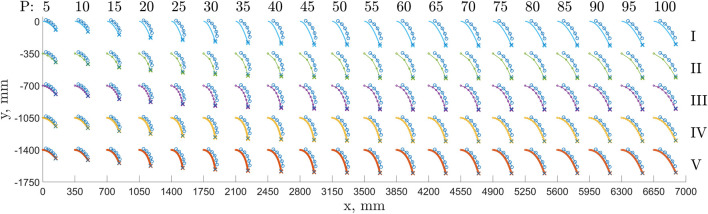
The figure shows modeled beam shapes for: the Euler-Bernoulli model (color: bright blue, row: I), the 4-node PDER model (green, II), the 8-node PDER model (purple, III), the 16-node PDER model (yellow, IV), the 32-node PDER model (red, V) for a range of internal pressures. The internal pressure ranges: **(A)** *P* = (5, 10, … , 50) psi **(B)** *P* = (55, 60, … , 100) psi.

Our measurements also included dynamic and steady-state positions of markers placed along the EFAM longitudinal axis. This data allows us to qualitatively evaluate the shapes of the EFAM when compared to our models. To do that, Figure 7 was compiled which holds the experimental data overlaid on top of our models at each internal pressure.

The figure shows experimental shape markers steady-state positions (blue-circle curve) and the end tip position for each case (blue cross). The figure also shows modeled beam shapes for: the Euler-Bernoulli model (color: bright blue, row: I), the 4-node PDER model (green, II), the 8-node PDER model (purple, III), the 16-node PDER model (yellow, IV), the 32-node PDER model (red, V) for a range of internal pressures. To account for the collective form of the plots, each consecutive model data set was shifted down by 350 mm down and right by 350 mm for a five psi pressure increment. The experimental data (blue) showing the tip position and the marker positions at the steady state is overlaid for each modeled beam.

It was observed that the Euler-Bernoulli beam model did not capture the EFAM curve accurately for the pressure range of 20–50 psi. This pressure range corresponds to low bending stiffness of the EFAM specimen. The curvature of the Euler-Bernoulli beams was smaller after a point when compared to the experimental data. Similar behavior was observed for the 4-node PDER model, however, the shape accuracy improved with each increase in the number of nodes.

Another important qualitative observation is that a similar trend of accuracy was seen for the rotation of the tip of beam. The tip rotation of the Euler-Bernoulli beam for the shape-misrepresented pressure range appears to be different than that which would be extrapolated from the last shape marker to the tip. Again, the tip rotation accuracy appears to improve with the number of nodes in the PDER models. This observation has implications for multi-section EFAM-based continuum soft robots as with each erroneous rotation of a section end, the error would affect errors in positions and orientations of consecutive sections.

### Natural Frequency and Damping

The dynamic properties were obtained based on the Cartesian coordinate data of the tip deflection. The static bias in the time histories of the coordinates was cancelled with their steady-state values. Then based on the *z* (*t*) coordinate history for each subtest the damping ratio was calculated with the method described in the modeling section.

**FIGURE 8:**
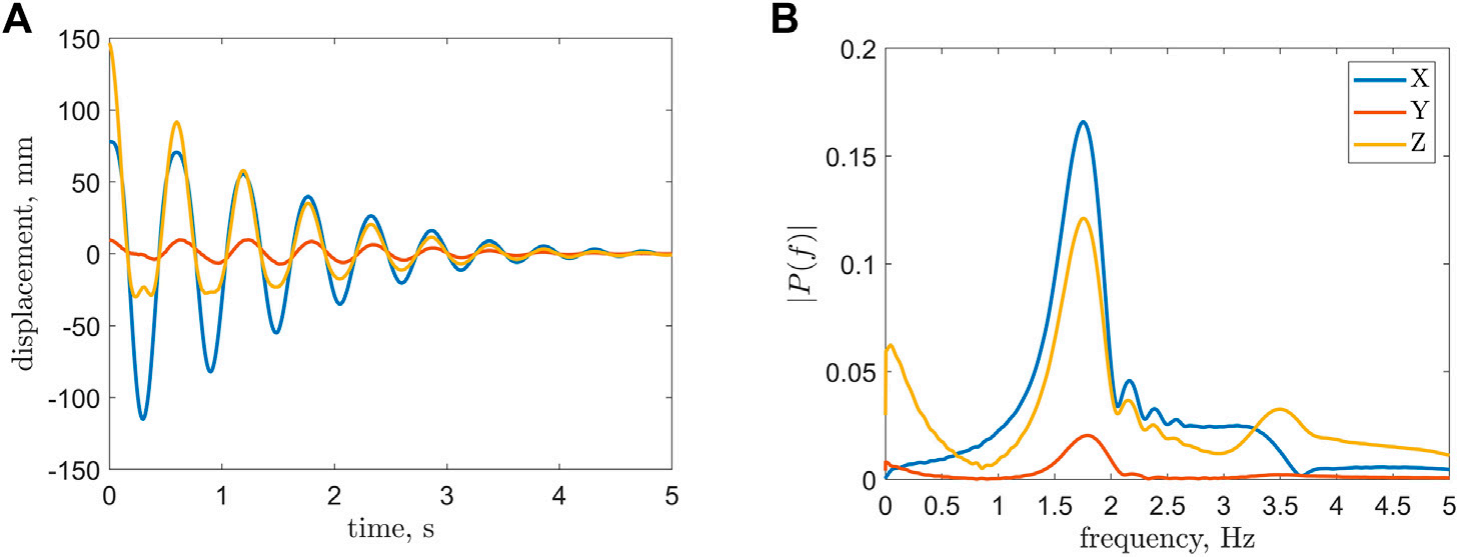
The figure is a visual example of the frequency analysis for a single test of an EFAM at an internal pressure of 15 psi. **(A)** A Relative Tip Displacement Trajectory. **(B)** Amplitude Spectrum of the Tip Displacement.

Damping ratio values were obtained for each tested internal pressure level with a visual example of a subtest at 15 psi demonstrated in Figure 8. It can be observed that there exists a slight bump in the *z*(*t*) time history. The bump and the asymmetry of the signal comes from large displacement of the tip. The deflection is so large that the tip rotates more than 90°. At the peak of its trajectory, the tip travels upwards. Fortunately, our method for finding the damping ratio operates only on the upper peak values and the bump does not affect the algorithm. This should also remind the reader that the EFAM was a non-linear highly-flexible beam, and that approximating it with a linear Euler-Bernoulli model was an attempt to obtain an approximation useful for engineering purposes.

**FIGURE 9:**
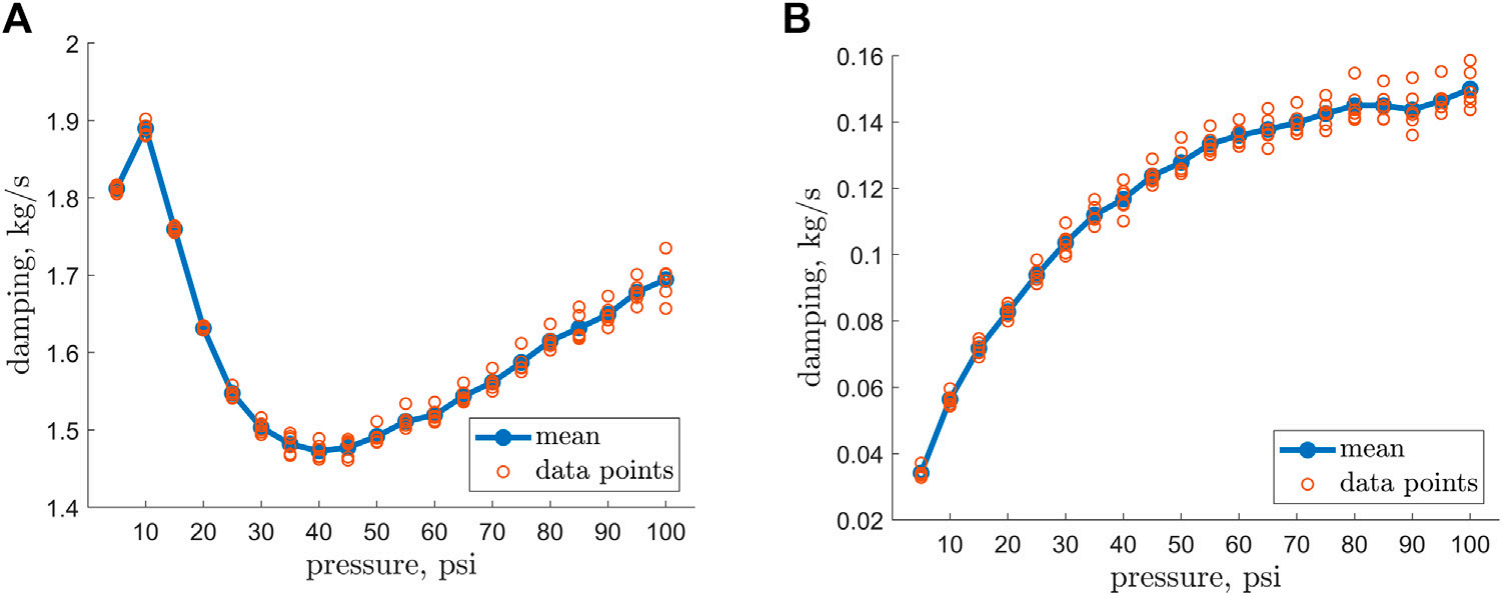
**(A)** Natural frequency versus EFAM internal pressure. **(B)** Damping ratio coefficients versus EFAM internal pressure.

The damping-pressure trend appears to be strongly dependent on internal pressure (Figure 9). A fourfold increase in damping was observed in the operating range of internal pressure. The natural frequency of the EFAM turned out to have a strongly non-linear trend with the frequency dropping from low pressures to 40 psi and linearly raising for larger pressures (Figure 9).

The experimental data was employed for determination of the dynamic properties such as damping and natural frequency. From a robotics perspective, knowledge of the minimum natural frequency is critical for avoiding structural resonance when controlling a soft robot that bends. The damping values can additionally help to estimate how fast the robot would stabilize. For example, with our result in Figure 9, it can be reasoned that for a continuum robot composed of more than one EFAM, the lower bound on the robot natural frequency will be greater than the lowest natural frequency of an EFAM.

Interestingly enough, in Figure 5 and Figure 9 minima exist at internal pressures of 25 psi for the bending stiffness and 40 psi for the natural frequency. Typically, when characterizing beam samples of different stiffness, one could expect that the beam with the minimum stiffness would also have the minimum natural frequency. However, this is not the case in our results. A possible explanation for this is that the natural frequency of a beam also depends on the beam length (Alper Erturk, 2011)). As it can be observed in Figure 4, the length increases non-linearly with an increase of internal pressure. Therefore, the sudden increase in length contributes to higher natural frequency, and therefore to the positive shift of the minimum when compared to the results for the bending stiffness.

### Limitations

Here, we enumerate study limitations.

1) Only one EFAM was tested, and it remains to be seen if an EFAM of the same diameter but different length would yield the same result.
2) The EFAM diameter was observed to slightly vary along the length, so that the beam cross-section is not uniform.
3) The bending stiffness value was only obtained for one loading condition. It is unclear whether the bending stiffness is dependent on the amount of EFAM deflection.
4) The bending stiffness was always measured under the assumption that the EFAM was at free strain for the Euler-Bernoulli model. Its agreement with the PDER model which included extendable links showed that the axial strain was indeed negligible for this experiment. However, how the bending stiffness changes with the actuator axial strain remains unknown.
5) A non-linear behavior is observed for pressures up to 40 psi in every calculated property. The effect and its potential relation to the FAM’s braid angle need further investigation.

Despite limitations of this study, methods for determining EFAM bending properties were established and provided physical insights into EFAM bending behavior.

## Conclusion

An extensile fluidic artificial muscle (EFAM) was analyzed for its bending properties over a relatively large range of pressures. Motion of the cantilevered testing specimen, an EFAM muscle of a blocked force of 300 N (at 75 psi) was captured and its measured bending properties were assessed: bending stiffness, natural frequency, and damping.

EFAM bending stiffness increased more than four times from internal pressure of 25 psi to 100 psi. This result is important for modeling behavior of continuum soft robots. A common trend in the literature is to characterize the axial properties of an EFAM (Tondu and Lopez (2000); Liu and Rahn (2004)), and then to determine Young’s modulus so that it could be used to calculate bending moments of the muscles (Trivedi et al. (2008)). This approach enabled modeling of continuum soft robots and reduced error when compared to the previous purely kinematic constant-curvature models (Webster and Jones (2010)). However, as observed in our results (Figure 5), bending stiffness is strongly and non-linearly dependent on EFAM internal pressure. Therefore, our results can lead to improved modeling of bending and helical FAMs (Guan et al. (2020)), as well as continuum soft robots that use FAMs for intrinsic actuation.

The natural frequency of the cantilevered EFAM changed by 20% from a minimum at 30 psi and a maximum at 10 psi (Figure 9). This gives interesting insight from the robotics perspective. The natural frequency increases with stiffness, so that a continuum soft robot composed of more than one EFAM should have a natural frequency greater than that measured for one EFAM (if the mass properties stay the same). Our results thus yield a lower bound on natural frequency of EFAM-driven continuum soft robots. Knowledge of this bound is important for designing control systems that avoid excitation at rates that can induce structural resonance. The damping values of the EFAM specimen increased by 450% from a minimum at 5 psi and a maximum at 100 psi. This provide insight on the stabilization of a continuum soft robot depending on its internal pressure. Due to a steep increase in damping for low pressures, if the robot operated at least at pressures higher than 30 psi, it would achieve two thirds of its maximum operational damping.

Changes to EFAM internal pressure strongly affect the bending properties of the muscle. The properties such as bending stiffness and damping can change their values fourfold. The natural frequency can also change by 20%. These strong relationships of bending properties to the internal pressure are recommended to be taken into account when designing structure and control of an EFAM-driven continuum soft robots carrying payloads.

## Future Work

In the future work, a broader set of FAM specimens should be examined including both extensile and contractile fluidic artificial muscles (CFAMs), and attempt at modeling efforts which would match the axial properties as well as the bending properties. The modeling efforts could be validated against different loading cases. Next steps could include experimental validation of the experimental values in static and dynamic models of EFAM-driven continuum soft robots.

## Data Availability

The raw data supporting the conclusion of this article will be made available by the authors, without undue reservation.
